# Parent–Toddler Behavior and Language Differ When Reading Electronic and Print Picture Books

**DOI:** 10.3389/fpsyg.2017.00677

**Published:** 2017-05-16

**Authors:** Gabrielle A. Strouse, Patricia A. Ganea

**Affiliations:** ^1^School of Education, Division of Counseling and Psychology in Education, University of South Dakota, VermillionSD, USA; ^2^Language and Learning Lab, Department of Applied Psychology and Human Development, Ontario Institute for Studies in Education, University of Toronto, TorontoON, Canada

**Keywords:** shared reading, e-books, toddlers, parent–child interaction, media

## Abstract

Little is known about the language and behaviors that typically occur when adults read electronic books with infants and toddlers, and which are supportive of learning. In this study, we report differences in parent and child behavior and language when reading print versus electronic versions of the same books, and investigate links between behavior and vocabulary learning. Parents of 102 toddlers aged 17–26 months were randomly assigned to read two commercially available electronic books or two print format books with identical content with their toddler. After reading, children were asked to identify an animal labeled in one of the books in both two-dimensional (pictures) and three-dimensional (replica objects) formats. Toddlers who were read the electronic books paid more attention, made themselves more available for reading, displayed more positive affect, participated in more page turns, and produced more content-related comments during reading than those who were read the print versions of the books. Toddlers also correctly identified a novel animal labeled in the book more often when they had read the electronic than the traditional print books. Availability for reading and attention to the book acted as mediators in predicting children’s animal choice at test, suggesting that electronic books supported children’s learning by way of increasing their engagement and attention. In contrast to prior studies conducted with older children, there was no difference between conditions in behavioral or off-topic talk for either parents or children. More research is needed to determine the potential hazards and benefits of new media formats for very young children.

## Introduction

Researchers have long acknowledged the importance of children’s environment in their language development (Hart and Risley, 1995; Snow, 1983). Shared book reading is one activity that can be particularly supportive of language development. Shared reading with preschoolers is linked with language growth and emergent literacy skills (National Early Literacy Panel, 2008; Sénéchal et al., 2008); and infant–caregiver reading is predictive of vocabulary growth (Debaryshe, 1993; High et al., 2000; Karrass and Braungart-Rieker, 2005).

Electronic books also carry some literacy benefits (Zucker et al., 2009; Takacs et al., 2015). Research on early versions of electronic books, such as CD-ROM books played on computers, shows that preschool and elementary children learn important literacy skills from electronic books, including phonological skills (Chera and Wood, 2003; Littleton et al., 2006; Shamir and Korat, 2007), vocabulary (Segers and Verhoeven, 2002; Shamir and Korat, 2007; Ihmeideh, 2014), print awareness (Ihmeideh, 2014), word reading (Shamir and Korat, 2007; Segal-Drori et al., 2010) and story comprehension (Doty et al., 2001). Because of the extra features they incorporate, such as built-in dictionaries and animations of story events, electronic books may support the development of literacy skills to an even greater extent than books without these enhancements (Rehbein et al., 2002; Verhallen et al., 2006; Korat and Shamir, 2008, 2012). A recent meta-analysis concluded that electronic books support story comprehension and vocabulary gains beyond that provided by print books (Takacs et al., 2015). However, electronic book studies have focused on pre-readers, early readers, and readers (ages 3 and up). Literacy benefits to infants and toddlers may differ.

One important mechanism by which shared reading with pre-readers impacts language development is through the adult–child interactions that take place during reading (Mol et al., 2008). If electronic books serve to disrupt the interactions that adults and young children have during reading, that may play a detrimental role in the literacy development of very young children. There is reason to believe that important differences exist in the way parents and children interact with new technologies and traditional formats (Chiong et al., 2012; Parish-Morris et al., 2013; Krcmar and Cingel, 2014; Willoughby et al., 2015). In the current study, we extend the literature on parent–child picture-book reading by investigating the impact of the book’s medium on the language and non-verbal behaviors parents and their 17- to 26-month-old children use during reading. We also take steps to address whether differences in parent and child behavior and talk during reading may be linked to differences in learning new information from the picture book. We first review prior research on traditional picture-book reading with this age group to reveal adult and child behaviors during reading which may impact learning and then present the emerging literature on shared reading in digital formats. Taken together this research informs our hypotheses regarding potential medium-related differences in parent–child reading behaviors.

### Shared Reading with Print Picture Books

To identify parent and child behaviors important to learning in reading contexts with our target age group, we reviewed the literature on shared reading with children under the age of 3. Two main categories emerged: non-verbal behaviors and parent–child talk. Parent and child behaviors in these categories vary in response to the age and linguistic growth of children. According to this research, parents of children under 18 months use both verbal and non-verbal attention-grabbing techniques and provide many labels during reading (DeLoache and DeMendoza, 1987; Sénéchal et al., 1995; Martin, 1997). They often point and ask simple questions, and interactions may be comprised of simple linear turn-taking (Sénéchal et al., 1995). This contrasts with parents of older toddlers and preschoolers who rely less on non-verbal behaviors and labeling to direct attention and use more complex speech in more extended reciprocal interactions (DeLoache and DeMendoza, 1987; Goodsitt et al., 1988; Sénéchal et al., 1995; Martin, 1997).

#### Non-verbal Behaviors and Affect

Unfortunately we found no literature directly linking non-verbal behaviors with literacy growth. However, because non-verbal behaviors play an important role in attention directing, they may be influential in children’s language learning. DeLoache and DeMendoza (1987) reported that infants often used pointing during reading to initiate interactions with their parents, especially at 15 months. Mothers interpreted their infants’ points as requests for information and generally provided a label. Murphy (1978) observed that pointing during reading was often accompanied by a verbal label from the mother when children were 14 months, but that by 20 months mothers instead asked children to provide the label for the referent. Thus, pointing may initiate and direct interactions in which language learning occurs.

Additionally, young children’s engagement in the reading process may be enhanced by giving them control to turn the pages of the book. Observations of parents and infants indicate that infant page turning increases as infants approach 12–14 months, is quite popular through the second year of life, and decreases in frequency around 24 months (Murphy, 1978; Martin, 1997; Loeb et al., 2015). Goodsitt et al. (1988) also reported a decrease in child page turns between 2 and 3.5 years of age. Murphy (1978) argues that once children have mastered the page-turning activity they shift their focus to looking at the pictures in the book. Goodsitt et al. (1988) add that mothers may encourage young children to practice page turns as part of learning the “rules” of reading. In addition, younger children may be more reliant on physical actions to maintain engagement in reading. Thus, we include both pointing and page turns as potentially important behaviors that may enhance toddlers’ shared reading experiences.

The emotional quality of the reading interaction may also play a potential role in supporting learning from shared reading. Research with preschool and elementary children indicates that the affective quality of reading interactions predicts children’s motivation for reading (Sonnenschein and Munsterman, 2002), frequency of reading (Leseman and de Jong, 1998; de Jong and Leseman, 2001), quality of parent language during reading (Leseman and de Jong, 1998; de Jong and Leseman, 2001), and children’s emergent reading skills (Bingham, 2007). Research on the emotional quality of the reading interaction with younger children is limited and suggests a complex interaction with cultural variables and reading styles (Cline and Edwards, 2013, 2017). However, because of its importance in older groups we decided to include a measure of child affect in our study.

#### Parent and Child Talk

Parent language, especially talk that is adaptive based on the developmental level of the child and results in increased child talk, is an important component of reading interventions that are successful in increasing preschoolers’ language acquisition (e.g., Whitehurst et al., 1988). Many have argued that the progression of parent language from simple to more complex is supportive for younger children’s language development as well (e.g., DeLoache and DeMendoza, 1987; Goodsitt et al., 1988). However, specific information about the best language to use when reading with children ages 2 and under is an area open for further study.

Two studies have indicated that particular types of parent language are associated with more talk on the part of their young co-readers. Sénéchal et al. (1995) noted that 9-, 17-, and 27-month-olds talked more when parents asked more questions and provided more feedback. Fletcher and Finch (2015) also found that 2-year-olds were more responsive when they were asked questions and received positive feedback, at least when reading non-narrative text. In addition, toddlers responded more when parents used more verbal attention-getting statements. Thus, questions, feedback, and attention-getters may be beneficial, but more research is needed to establish causal directionality and links with child language growth.

#### Links with Learning

The non-verbal and verbal behaviors reviewed above have not been directly linked to toddlers’ language learning, but have been shown or predicted to increase engagement with reading by way of increased verbal and non-verbal participation and attention to the book. Overall child attention and engagement during reading *has* been linked to developmental benefits. Children’s verbal and non-verbal responses during reading at age 2 predicted their language ability at 2.5 and 4 years (Crain-Thoreson and Dale, 1992), and 14-month-olds’ verbal and non-verbal responses, rated interest, and time spent reading predicted language development at 18 months (Laakso et al., 1999). Fletcher et al. (2005) observed children repeatedly between age 18 and 24 months and found high stability in individual children’s responsiveness (verbal and non-verbal participation) to reading and joint attention to the book across sessions. There was also a correlation between children’s attention and their vocabulary at 24 months. Thus, it is possible that attention and engagement partially mediate the path between the non-verbal and verbal behaviors identified above and toddlers’ language acquisition during reading.

In the current study, we add to the literature on traditional parent–child picture-book reading by reporting measures of parent and child non-verbal and verbal behaviors similar to those reviewed above. We extend the literature by also incorporating a vocabulary learning outcome. A growing number of studies have shown that by 18 months children learn specific words presented to them during a picture-book reading interaction (Ganea et al., 2008; Tare et al., 2010; Horst et al., 2011; Walker et al., 2013). We included a test of learning of a specific word presented in the book to assess whether language learning occurred during the particular parent–child reading session and with the goal of answering whether any measured parent and child behaviors were mediators of this learning.

In summary, research on shared reading of print books has lead us to identify both non-verbal behaviors (pointing, page turns, child affect) and aspects of parent and child language (amount and content of parent and child talk) that may serve to increase toddlers’ learning during picture book interactions. In the current study we observe these variables during a parent–child reading session with either print- or electronic-format books. Our goal is to document format-related differences in these behaviors, as well as potential links between the identified behaviors and children’s learning. In the next section we review the emerging literature on shared reading with electronic books to inform our hypotheses regarding potential format-related differences in behavior and learning.

### Shared Reading with Electronic Books

Electronic books include a number of enhancements that may lead to different parent and child behaviors and child learning than print books. For example, many electronic books read themselves and include animated pictures and games. Research with preschoolers and kindergarteners has addressed the pros and cons of including digital scaffolding, picture cues, read-alouds, highlighted text, word pronunciations, built in dictionaries, and other features (see Moody, 2010; Takacs et al., 2015). Takacs et al.’s (2015) meta-analysis revealed that multimedia features like animations and sound effects were supportive of vocabulary and story comprehension, whereas built-in games and hotspots (spots on the screen that lead to an on-screen event when activated) detracted from learning. Studies have not yet addressed how most of these individual features influence adult–child interaction, but one meta-analysis suggests that multimedia features, taken together, may be equally effective for children’s learning as scaffolding by an adult (Takacs et al., 2014). The authors argue that “multimedia elements provide scaffolding of children’s understanding and word learning that is comparable to adult scaffolding during storybook reading (p. 10).” Thus, it is possible that multimedia books afford less parent–child talk because the child is focused on and learning from the narration and animation in the book, rather than scaffolds from a parent.

A few studies have investigated the role of the electronic format on adult and preschooler behaviors while reading. Moody et al. (2010) found that 3- to 6-year-olds in Head Start classrooms labeled more pictures when they were read a print book than when they read the same book in electronic format. Children also tended to label more pictures when their hotspot usage in the electronic book was restricted than when they were free to activate as many hotspots as they desired. Other types of child talk did not differ based on medium, but labeling was one of the most frequent ways in which children initiated communication with their co-reader, a trained research assistant. In this study children were most communicative when reading print books and least communicative when reading electronic books with many distracting hotspots, possibly indicating that hotspots drew their attention away from their in-person interaction. In a more recent study, 3- to 5-year-olds who read electronic and print storybooks made a similar number of overall utterances with both book types, but made more story-related references with print books and more comments about the book/device itself with the electronic books (Richter and Courage, 2017).

Three recent studies with preschoolers reading with their parents have resulted in similar findings regarding parent language. In three different studies, parents were observed reading electronic or print books with their children and children were tested on their story comprehension. In all three studies, there was evidence that parents reading electronic books spent less time talking about story-related content and more time on off-topic (usually device-related) talk than parents reading print stories (Chiong et al., 2012 – 3- to 6-year-olds; Krcmar and Cingel, 2014 – 2- to 5-year-olds; Parish-Morris et al., 2013 – 3- and 5-year-olds). There was also evidence that children’s story comprehension was lower when reading electronic books with all groups except Parish-Morris and colleague’s older group, who reached ceiling on the comprehension measure. The authors argued that one reason for the lower comprehension scores may have been the lower quality of parent language during reading. This was the case both with electronic console books (Parish-Morris et al., 2013) and iPad books (Chiong et al., 2012; Krcmar and Cingel, 2014).

One important difference between these three similar studies did arise: Chiong et al. (2012) reported that the reduction in content-related talk (compared to print books) was only present when parents read an enhanced e-book with hotspots with their children, and not when reading a basic electronic version in which no hotspots were present. Similarly, there was no reduction in comprehension for the basic electronic book, although there was an increase in non-content-related talk (again, compared to the print book). This suggests that the addition of interactive features to the book was what distracted parents and children from the story, not the device itself. On the other hand, Krcmar and Cingel (2014), using a basic book without hotspots, reported a decrease in content-related talk, increase in non-content-related talk, and decrease in comprehension with electronic compared to print books. Interestingly, Krcmar and Cingel also reported a negative relationship between prior electronic book experience and children’s comprehension of the electronic book. They suggested that children with more experience with iPads may view them as toys and invest less mental effort in learning from them. If true, an increase in the prevalence of home iPad use between 2012 and 2014 could partially explain the discrepancy in the two studies’ findings.

None of these studies have reported parent and child talk with electronic books in children under the age of 2. However, in one study parents of 1- to 4-year-olds self-reported that they less frequently labeled items in stories or stopped to discuss stories when reading electronic books with their children than when reading print, and that their children were less likely to label items in electronic stories or tell back parts of the story (Strouse and Ganea, 2017a). These reports appear to be consistent with the findings regarding parent–child talk observed in older samples.

Parents in Strouse and Ganea’s (2017a) study also reported that they and their children were less likely to point when reading electronic than print books. Differences in pointing and other non-verbal behaviors may be afforded by the different media platforms as well. For example, one study of 3- and 4-year-olds in classrooms indicated that children who were able to hold an electronic device during reading were more likely to look at and touch the device whereas those who did not hold the device were more likely to gesture (Roskos et al., 2012). If infants are likely to be holding the device on which they are reading they may be less likely to point and more likely to touch the device. In another study with 4-year-olds, children were more likely to physically control an electronic than a print book when reading with their parents (Lauricella et al., 2014). Thus, differences in how the parent–child pair hold electronic versus print books may result in format-related differences in gesturing. In addition, the physical action need to turn an electronic page requires a touch rather than a physical flip. Because tapping is a simpler motor movement it may be more easily available to infants and toddlers than print-book page turns.

Despite reports that content-related talk and physical gesturing are infrequent there is evidence that an electronic format is more engaging for children. Richter and Courage (2017) reported that 3- to 5-year-olds stated a preference for the electronic books over the print books in their study. Chiong et al. (2012) reported that their 3- to 6-year-olds were more engaged with both types of electronic books they used (enhanced and basic) than print books. Moody et al. (2010) reported that a group of Head Start preschoolers who read an electronic book with an adult maintained participation in reading longer than a group who read a print book. Finally, Verhallen and Bus (2009) found that 5-year-olds with low language skills invested more mental effort across multiple readings when books were animated rather than comprised of static images, suggesting that enhancements available in electronic formats acted to maintain interest in reading. We know of no studies measuring the interest level of children younger than 3, but expect that children in this age group will find touchscreens a particularly engaging medium because they are so effortless for young children to control.

Based on research with print books, we expect that parent–child talk in our age range (17–26 months) will consist of exchanges of fairly low complexity that are focused on simple labeling and pointing rather than multifaceted connections. Device-related talk may not impair learning from these kinds of low-complexity interactions because they do not depend on drawing connections across multiple story aspects. However, any differences in the amount of pointing and labeling between the two formats (as parents reported in Strouse and Ganea’s, 2017a) survey may influence word learning because children in this age range often rely on adult referential cues like pointing to identify the referent of new words (Baldwin, 1993; Grassmann and Tomasello, 2010). It is also possible that animations in the electronic book would support children’s word learning in the absence of referential cues from an adult, as 18-month-olds have also been shown to use salience cues like illumination and movement when learning words (Moore et al., 1999). It remains to be seen whether animations could provide similarly supportive referential cues as adults for children in this age range.

Beyond differences in word learning resulting from the presence or absence of attention-directing cues, there is reason to suspect medium-related differences in learning even when these cues are matched. In Strouse and Ganea’s (2017b) study, 17- to 23-month-old children were read either an electronic or print book with no text, animations, or sounds in a short, scripted interaction with a researcher. Pointing and labeling were scripted and equivalent across conditions, and animations were absent. Children were then tested on a new word presented in the book. Toddlers displayed more transfer and generalization of the newly learned word when they were read a print rather than electronic version. The authors hypothesized that this difference may have been a result of expectations children brought to the learning situation built on prior experience with the formats (Strouse and Ganea, 2017b). Thus, there is reason to expect that even well-matched books may result in differences in learning.

### Research Hypotheses

Analyses will be conducted to address the following hypotheses:

H1: Parents will produce less pointing and content-related language and more off-topic and behavior-related language with electronic than print books.
H2a: Children will produce less pointing and child-initiated content-related language with electronic than print books. Children with prior experience with e-reading may produce less content-related talk when reading in the electronic format than those without experience.
H2b: Children will exhibit higher levels of attention and engagement with electronic than print books. Because of increased engagement, we also expect children will display higher levels of positive affect with electronic than print books.
H3: Children read electronic books will display less learning than those read print books. Prior experience with electronic books will be associated with lower learning from electronic books, but play no role in learning from print books.

Finally, in this study we are interested in the role that parent and child behaviors during reading play in mediating the relationship between book format and learning. Potential mediators will be identified from behaviors with large format-based effects.

## Materials and Methods

### Participants

Participants were 152 children aged 17.0–26.9 months (*M* = 21.33, *SD* = 2.90; 77 male) from Toronto, Ontario and surrounding areas. They were recruited through advertisements, local street fairs, child care centers, and the local Science Centre. One hundred and two of these children were randomly assigned to the two experimental conditions: 50 were read electronic books and 52 were read print books. The remaining 50 children were randomly assigned to two control groups: 25 in electronic format and 25 in print format. Children in the control conditions did not read books but were tested on the learning outcome. Ten additional children were not included in the analyses due to unwillingness to participate in the procedure (8), having the book at home (1), and technical difficulties with the recording (1). Children who participated had no developmental delays and were exposed to English at least 50% of the time.

This study was carried out in accordance with the approval of the University of Toronto Research Ethics Board and written informed consent was obtained from all parents. The final sample was identified by their parents as 67.8% White and 19.7% mixed ethnicity. The remaining participants were identified as Asian (9 children, 5.9%), African–Canadian (2 children, 1.3%), and “other” ethnicity (3 children, 2.0%). Five parents (3.3%) opted not to respond. Parents were generally well-educated, with a median and modal response of a 4-year university degree.

### Materials

#### Picture Book

Children in the experimental conditions were randomly assigned to be read either electronic or print versions of two 10-page picture books by their parents. The electronic books were commercially available by a major worldwide book publisher. The app containing the books was listed as “educational,” including claims such as, “Helps to develop hand-eye coordination and focusing skills in young babies,” and, “Helps older babies and toddlers with language acquisition.” Each book introduced four animals in two-page sequences. The first page featured an adult animal of a species and the second page introduced the baby animal by name (e.g., joey for a baby koala). We chose two books, one which presented farm animals (sheep, duck, horse, cow) with which parents reported most children were already familiar, and one which presented wild animals (lion, zebra, koala, crocodile) with which parents reported most children were less familiar. Both books also included two final pages including a vehicle and a human baby.

The electronic book included background music, animation, and sound effects for each page as well as an automatic voiceover that read the text. The text was comprised of 1–2 sentences per page with 3–4 words per sentence (e.g., Hello, fuzzy ducklings!). The animations and sounds played automatically as each page was turned, and there were no actions or hotspots for parents and children to tap for extra features. A tap was required to turn each page, and this was the only action that produced a contingent response.

The publisher of the electronic book has a similar line of printed board books with very comparable content and illustrations, however, we could not find an exact printed match for the electronic book. As a result, our print book was created by taking screenshots of each page of the electronic book. These were printed, laminated, and bound. Books were printed to be the exact size they appeared on the tablet screen. Children in the two control groups were not read any of the books.

#### Test Items

All children (experimental and control) were tested on their receptive understanding of two animal names. At the beginning of the session, parents were given a checklist of 16 animal names – 8 that were presented in the two books used in this study along with 8 animals from other books from the same published set. Parents were asked to identify which animal names their child understood, understood and said, or did not know. Based on these parent reports we selected animals individually for each child to use for testing.

To test for word learning from the book, we chose an *unfamiliar target* randomly from among the child’s unknown animals from the wild animal book (lion, koala, zebra, crocodile). We then chose two distractor animals: another from the wild book (*seen distractor*) and an *unseen distractor* from a book the child did not read, either a seahorse or a whale. Children who did not have enough unfamiliar animals to create this set of three animals to be tested with were excluded from the analyses related to learning. Sixty-three children – 42 experimental (20 who read e-books, 22 who read print books) and 21 control – were retained for these analyses.

Each child was also tested with a familiar set of animals as an indicator of the child’s understanding of and compliance with the testing procedure. For each child’s familiar animal testing, we randomly chose a *familiar target* from among each child’s known animals from the farm book (sheep, duck, horse, cow). We then chose two distractor animals from the child’s remaining known animals, one *seen distractor* from the farm book and an *unseen distractor* animal, either a frog or a bird. For all choices, names the child said were prioritized above those the child understood but did not say.

Children were asked to identify each target animal (familiar and unfamiliar) three times: using a cartoon drawing of each target animal taken from screenshots of the book, with a photograph of each real target animal, and with small plastic replicas of the animals. For the two-dimensional trials (cartoon and photograph), children in the print book conditions were tested with laminated cards of the animals and children in the electronic book conditions were tested with the same pictures on the tablet screen. The images appeared the same size on the cards and on screen. Children in all conditions were tested with the same replica animals.

#### Questionnaires

Parents were asked to fill out the MacArthur-Bates Communicative Development Inventory Short Form Level II (Fenson et al., 2000). This measure is comprised of a checklist of 100 words for which parents indicated whether their child said each word. Children had vocabularies around average for their age. Parents checked an average of *M* = 41.0 words (*SD* = 25.9) on the MacArthur-Bates checklist (percentile: *M* = 44.1, *SD* = 29.0).

Parents were also asked to fill out a questionnaire which included demographic information for the family and information about their child’s exposure to English, their child’s knowledge of the animal names in the books, and their experiences with shared picture book reading, electronic books, videos, and other media.

Parents of 21% of children reported that they had prior experience with e-books. Of the parents who reported each activity, parents estimated that their children were read traditional print books an average of 5.6 h per week (*SD* = 4.36), read e-books 1.29 h per week (*SD* = 1.88), watched 3.43 h of videos and television (*SD* = 4.32), and played 1.01 h of apps and games (*SD* = 1.82).

### Procedure

The experimenter began by warming up with the child by playing on the floor with puzzles or other toys (on campus) or at a child-sized table with stickers and coloring materials (at the Science Centre) while the parent completed the questionnaire and vocabulary checklists. Once the child was comfortable, the child and parent were invited to the testing area.

#### Reading

Children and parents were encouraged to sit however they felt most comfortable for reading. This included sitting in an armchair with the child on their lap, sitting at a child-sized table next to the child, sitting together on the floor, and other positions.

Parent–child pairs were randomly assigned to participate in one of two experimental conditions (print or electronic book) or in one of two (print or electronic) control conditions^1^. Half of parents in each experimental condition were asked to read the farm book first; half read the wild book first. After setting up a camera and audio recorder, the experimenter left the room to allow parents and children to read without distraction. She returned to the room when she heard that the pair had finished reading. Parent–child pairs participating in the control conditions did not read the book and participated only in the following test.

#### Test

Parents and children were not aware that children would be tested on the animal names until after they finished completing the animal checklist and, in the experimental groups, completed reading the books. The experimenter began by exclaiming, “Now I need your help to find some animals!” For each of the test trials she presented the children with the three animal pictures or replicas, allowed the child to touch them if they wanted, and then asked the child to “Show me the [target]!” Once the child made a choice, the experimenter replied, “Thank you!” and continued to the next trial. There were six total trials (three with familiar animals and three with unfamiliar animals). The order of the two sets of animals (familiar and unfamiliar) and two picture formats (cartoon images taken from the book and photographs of real animals) were counterbalanced. The two sets of replica animals were always presented last. Within each set, the unseen distractor was always placed in the center. The target and seen distractor alternated in the left and right positions.

#### Transcription

Parents and children’s reading sessions were transcribed from video using CLAN^2^. In four cases the original videos were lost and sessions were transcribed from audio recordings. Transcription began when parents opened the print book or tapped the icon to begin the electronic book. Transcription ended when the book was closed.

#### Coding

All coders were blind to study hypotheses.

##### Non-verbal tactile behaviors

Parents’ and children’s book-related tactile behaviors were coded offline using the Datavyu program^3^. Two coders reviewed the videos and recorded the number of times children and parents pointed at the book or turned the pages of the book. Reliability for 21% of the sample, measured by the intraclass correlation coefficient, was *r* = 0.90 for child points, *r* = 0.91 for child page turns, *r* = 0.93 for parent points and *r* = 0.95 for parent page turns.

##### Child language

Coders reviewed transcripts of the parent–child reading sessions and assigned each child utterance to one of the following categories: book content-related talk (e.g., “moo”), book behavior-related talk (e.g., “turn page,” “touch”), and off-task comments. Book content-related talk was further broken into three categories: child-initiated comments, questions, and responses. The number of utterances in each category was summed for each child for each book. A second person coded approximately 50% of the videos. Interrater reliabilities, measured by the intraclass correlation coefficient, were: book-related comments, *r* = 0.83, book-related questions, *r* = 1.0; book-related responses, *r* = 0.89; book-related behavioral talk, *r* = 0.63; and off-task talk, *r* = 0.83. Questions were extremely rare and were thus excluded from analyses.

##### Parent language

Parent utterances were also assigned to categories from the session transcripts. Coders initially assigned parent speech to book-content-related speech, orienting talk (i.e., comments designed to redirect children’s attention to the book or to do book-related behaviors), direct reading of book text, or off-task talk. Book-content-related speech was further broken into questions, simple statements about things directly observable in the book, elaborations about book content that went beyond the information provided, negative or positive feedback given to the child, and simple repetition of child speech. The number of utterances in each category was summed for each parent for each book. Utterances were coded by a second individual for approximately 50% of the sample. Intraclass correlations were: questions, *r* = 0.96; simple statements about content, *r* = 0.91; elaborations, *r* = 0.62; feedback, *r* = 0.67; repetitions, *r* = 0.65; orienting/behavior-related speech, *r* = 0.81; reading, *r* = 0.96; and off-task speech, *r* = 0.90.

Patterns of parent content-related talk (i.e., questions, statements, elaborations, feedback, and repetitions) were consistent with what we expected in this age group (high numbers of simple statements and questions) and consistent across categories, so parent content-related talk was combined into a total score for analysis. Direct reading of text from the page of the book was coded into a separate category that was not included in our analysis of parent content-related talk. This was done, in part, to control for differences that resulted from the automatic narration in the electronic book.

##### Attention

Children’s attention to the book during reading was coded offline as a proportion of time their eyes were on the book while the book was open/on screen. Children who went off camera for less than 30 s during a book-read were given a proportion out of the total codeable time; those off-screen for more than 30 s were not coded. A second person coded 31% of the participants in the groups who read. Coders had an intraclass correlation of *r* = 0.89.

##### Global behaviors (engagement and affect)

Children’s availability for reading, their affect, and active participation were coded from video by two coders using Likert scales adapted from Deckner et al. (2006). The book reading sessions were partitioned into 30-s intervals and a code was assigned to each interval with at least 15 s of codeable time (child viewable on screen and book open for reading). Availability for reading was measured from 1 = child had less than 3 s during the interval in which they were present and attending to the book to 5 = the child was present and not looking away for at least 27 s during the interval. Affect was measured from 1 = child protesting or crying for at least 7 s during the interval to 5 = child laughing or smiling for at least 7 s during the interval. Active participation was measured from 1 = child made no contributions during the interval to 5 = child made 7 or more verbal or physical contributions including comments, gestures, and manipulations such as turning the pages or pointing. Interrater agreement for 20% of the sample, measured using a weighted Kappa, was *κ* = 0.89 for availability, *κ* = 0.70 for affect, and *κ* = 0.81 for active participation. Scores for the 30-s intervals were then averaged for a composite score for each scale for each book the child was read.

##### Animal choice (learning)

Children’s animal choices during the test trials were recorded by the experimenter as the child’s first touch after the question prompt. A second coder reviewed children’s choices from video. Reliability was *κ* = 0.78. A third coder resolved all discrepancies.

#### Missing Data

Two children in the reading groups were unwilling to read the farm book and were thus missing data for all variables for that book. Their data for the wild book was retained. Because children were sometimes out of range of the camera or their sessions were transcribed from audio, four children were missing data for non-verbal behaviors for both books, three additional were missing a total duration score for one of the books, and another eight were missing one or both attention scores. Because missing data resulted from poor camera angles or lost videos, there is no reason to suspect missing data was systematic. Because children were often only missing a score for one of the two reads, we purposefully chose an analysis strategy that would allow us to retain their other scores without needing to impute or replace the missing values.

## Results

### Preliminary Analyses

The number of children in each condition with prior exposure to e-books did not significantly differ, but this variable was used as a covariate in future analyses because we predicted it may influence children’s participation with electronic books. In addition, there were no significant differences in age, gender, parent education, or vocabulary level for children with or without prior e-book experience. There were also no significant condition differences in the number of hours per week spent with either type of book, video and television, or apps and games.

### Analysis Strategy

Analyses are reported in two main sections: parent and child behaviors during reading (hypotheses 1 through 2b) and child learning (hypothesis 3). Since vocabulary and duration of prior media exposure were similar across conditions, these variables were not included in future analyses. However, age and e-book exposure (as a dichotomous code) were retained, despite being similar across groups, because they were of interest as potentially predictive of children’s reading behavior and learning. We chose to use linear mixed models because they are well-suited to model repeated measures data and allow for maximum participant retention in the case of a missing data point (Cnaan et al., 1997). As such, the outcome measures that follow were analyzed using a linear mixed model with compound symmetry with book content (farm, wild animals) as a repeated effect and fixed effects for book format (print, electronic), prior book exposure, and age. Our specified model also included a fixed effect for the interaction between book format and prior book experience, as it was important to testing our hypotheses. In addition, we included a fixed effect for the interaction between book format and book content because children may become more responsive to books when they are re-read (Fletcher and Jean-Francois, 1998). Thus we believed there may be an effect of both familiar content and familiarity with the device that should be controlled for in the model. The duration of time spent reading was included in the model as a time-varying covariate except when it was the outcome. Due to the large number of statistical results generated by these models, effects relevant to our hypotheses and discussion are reported in the text; full reporting of the results including the control variables (content, duration, age) can be found in the Supplementary Tables 1–7 and an overview of group differences in outcomes is presented in Supplementary Figure 1.

### Parent and Child Behaviors during Reading

#### Duration

Parent–child pairs spent almost twice as much time reading the electronic books than the print format books, *F*(1,88.44) = 74.70, *p* < 0.001 (electronic *M* = 3:35, *SD* = 0:49; print *M* = 1:54, *SD* = 0:55). Because of the large differences in duration spent reading due to our main variable of interest (book format) we control for duration in all subsequent models.

#### Hypothesis 1: Parent Non-verbal and Verbal Behaviors

Consistent with our hypothesis, parents pointed more when reading print books than electronic books, *F*(1,118.80) = 15.40, *p* < 0.001 (electronic *M* = 11.80, *SD* = 11.02, print *M* = 19.38, *SD* = 13.87; **Table 1**).

**Table 1:** Unadjusted means and parameter significance for parent behaviors.

Fixed effects	Electronic		Print		Parameter significance	
	*M*	*SD*	*M*	*SD*	*F*	*p*
Points	11.80	11.02	19.38	13.87	15.40	<0.001***
Content-related utterances	41.92	15.21	24.89	13.97	1.78	0.185
Reading utterances	6.28	5.93	14.61	4.59	20.54	<0.001***
Behavior-related utterances	4.37	4.06	1.90	2.46	0.13	0.716
Off-topic utterances	9.12	7.83	4.72	6.00	0.56	0.457
Page turns	5.98	3.65	5.62	2.56	0.75	0.390

*^∗^*p* < 0.05, ^∗∗^*p* < 0.01, ^∗∗∗^*p* < 0.001.*

Contrary to our hypothesis, there was no significant effect of format on the number of parents’ content-related utterances (excluding reading the text). Parents read more of the text from the page when they were reading print format books, *F*(1,125.51) = 20.54, *p* < 0.001 (electronic *M* = 6.28 utterances, *SD* = 5.93; print *M* = 14.61, *SD* = 4.59).

Also in contrast to our hypothesis and what has been found with older children, there were no significant medium-based differences in parents’ discussion of behaviors related to reading or off-topic talk. We also saw no difference in parent page turns.

#### Hypothesis 2a: Child Verbal and Non-verbal Behaviors

Contrary to our prediction, children who were read the electronic books tended toward more pointing than those who read the print books, although this did not reach significance, *F*(1,115.13) = 3.62, *p* = 0.060 (electronic *M* = 4.62, *SD* = 4.24; print *M* = 1.58, *SD* = 2.58; **Table 2**). They also produced significantly more self-initiated content-related comments when being read the electronic format books, *F*(1,125.03) = 6.97, *p* = 0.009 (electronic *M* = 5.46, *SD* = 4.85, print *M* = 2.13, *SD* = 2.54). There were no significant predictors for the number of times children responded to their parent.

**Table 2:** Unadjusted means and parameter significance for child behaviors and engagement.

Fixed effects	Electronic		Print		Parameter significance	
	*M*	*SD*	*M*	*SD*	*F*	*P*
Points	4.62	4.24	1.58	2.58	3.62	0.060
Content-related comments	5.46	4.85	2.13	2.54	6.97	0.009**
Content-related responses	7.92	5.91	6.18	5.65	0.96	0.330
Off-topic utterances	3.74	4.28	2.50	3.63	0.76	0.386
Behavior-related utterances	0.81	2.26	0.59	1.16	3.61	0.060
Page turns	4.09	3.38	2.35	2.68	4.42	0.038*
Attention (%)	91.15	9.72	82.62	21.03	21.78	<0.001***
Availability for reading (max 5)	4.60	0.56	4.13	1.08	17.60	<0.001***
Positive affect (max 5)	3.53	0.45	3.29	0.53	12.85	<0.001***
Participation (max 5)	3.35	0.85	3.27	1.07	1.32	0.253

*^∗^*p* < 0.05, ^∗∗^*p* < 0.01, ^∗∗∗^*p* < 0.001.*

Consistent with hypothesis 2, there was no difference in children’s off-topic talk (about snacks, flipping light switches, etc.) when reading electronic or print books. After adjusting for covariates, our model indicated that children produced more behavior-related talk when reading the print format books, *F*(1,123.19) = 3.61, *p* = 0.060, but this did not reach a standard level of significance, and the unadjusted means did not display this pattern (electronic *M* = 0.81, *SD* = 2.26, print *M* = 0.59, *SD* = 1.16).

Also consistent with hypothesis 2, we found a significant interaction between prior experience and child language. Children with no prior experience with e-books made more comments when reading electronic books, *F*(1,95.76) = 3.96, *p* = 0.049 (without experience: *M* = 6.13, *SD* = 5.11; with experience: *M* = 3.77, *SD* = 3.83).

We were unable to make any direct predictions about children’s page turns based on prior literature. According to our observations, children reading the electronic book turned more pages *F*(1,117.27) = 4.42, *p* = 0.038 (electronic *M* = 4.09, *SD* = 3.38; print *M =* 2.35, *SD* = 2.68), than children who read the print books, even after controlling for reading duration.

#### Hypothesis 2b: Children’s Attention and Engagement

Consistent with our hypothesis, children’s overall attention was significantly higher to the electronic format, *F*(1,120.95) = 21.78, *p* < 0.001 (electronic *M* = 91.15%, *SD* = 9.72%, print *M* = 0.62%, *SD* = 21.03%; **Table 2**), even after controlling for the extended duration of the electronic reading sessions.

Also consistent with our hypothesis, children made themselves significantly more available for reading (present and attending) when they were read the electronic than the print-format book, *F*(1,116.10) = 17.60, *p* < 0.001 (electronic *M* = 4.60, *SD* = 0.56, print *M* = 4.13, *SD* = 1.08), and had significantly higher levels of positive affect when reading the electronic book, *F*(1,113.78) = 12.85, *p* < 0.001 (electronic *M* = 3.53, *SD* = 0.45, print *M* = 3.29, *SD* = 0.53). There was no significant effect of book format (electronic, print) on the global measure of participation.

### Hypothesis 3: Children’s Learning

To determine if children were more likely to learn animal names when participating in different conditions, we ran generalized estimating equations (GEEs) using a binomial distribution with a logit link function. In these models, choices on the test trials (using screenshots from the book, photographs of the animal, and replica animals) served as the repeated effect; age, book format, performance on the familiar animal trials (as a proxy for children’s understanding of and cooperation with the testing procedure) and prior experience served as fixed effects. We also included the interaction between book format and prior experience with e-books as a fixed effect. In these models we used an autoregressive covariance structure as we expected that as the test items became less similar to the learning situation, the correlation between measurements may decrease. Main effects of condition are reported here; more details are available in the Supplementary Table 8.

In the first model we also controlled for the duration spent reading the wild book (to control for the time children were exposed to the new animals). Because of the reading duration variable, this model could not include the control groups (who did not read). There was a main effect of book format, Wald χ^2^ (df = 1) = 7.36, *p* = 0.007 in the opposite direction of our prediction. Children who read the e-book made more correct choices [electronic *M* = 1.93 (of 3), *SD* = 0.88; print *M* = 1.28, *SD* = 1.07; **Table 3**]. This corresponds to a medium to large effect, *d* = 0.66. Also in contrast to our hypothesis, there was no prior experience by format interaction.

**Table 3:** Unadjusted means for learning outcomes.

	Electronic		Print		Effect size
Correct choices (of 3)	*M*	*SD*	*M*	*SD*	*d*
Experimental	1.93	0.88	1.28	1.07	0.66
Control	1.30	1.16	1.36	1.29	-0.05

*Effect size computed for electronic versus print comparison. ^∗^*p* < 0.05, ^∗∗^*p* < 0.01, ^∗∗∗^*p* < 0.001.*

Finally, to test whether children in the experimental conditions outscored children in the control conditions, we removed reading duration from the model. The resulting model contained only 21 control children (10 electronic, 11 print; see Test Items) and thus was very underpowered to detect condition differences. The resulting model had poor model fit (a change in QICC from 141.32 to 233.86, lower is better) and returned no significant effects (see Supplementary Table 8). The effect size for the comparison of total correct unfamiliar animal choices between control and experimental groups was *d* = 0.22 overall. However, as can be seen in **Table 3**, this effect is dampened by a lack of learning in the experimental print group. The effect size between the electronic groups alone is a moderate *d* = 0.61. With such a practically significant effect size, we do not believe it is appropriate to draw strong conclusions from the lack of statistical significance in the poorly fitting, underpowered model.

### Mediation

According to Fritz and MacKinnon (2007) our sample of children used in the learning analyses (20 who read e-books, 22 who read print books) would give us adequate (0.8) power to test mediation only when there were large correlations (0.59) between both the predictor (book type) and mediator and between the mediator and the outcome (learning). Initial correlation analysis indicated that the only two predictors that even approached this criterion (with correlations larger than 0.3) were availability for reading and attention to the book. Thus, we tested these two variables for mediation using Hayes’ PROCESS macro^4^. Both mediation models were run using only data from the wild animal book (from which the unfamiliar target was chosen) and included children’s age and the duration spent reading as control variables.

The relationship between book format and learning was mediated by children’s availability for reading. Children who read the e-book were more available for reading, *b* = -1.0126, *SE* = 0.4931, *p* = 0.0495. Availability for reading was a marginally significant predictor for learning, *b* = 0.4248, *SE* = 0.2110, *p* = 0.0542. A model with book format, availability, age, and duration as predictors accounted for approximately 25% of the variance in learning (*R*^2^ = 0.2529). Bootstrapping with 5000 samples estimated the indirect effect of book format on learning through availability was significant at the 95% confidence level, *b* = -0.4301, *SE* = 0.2645, CI = -1.1154, -0.0343, supporting the mediational hypothesis.

Similar results emerged when attention was used as the mediator, measured by the percentage of time children spent with their visual focus on the book. Book format was a significant predictor of attention, *b* = -0.2636, *SE* = 0.1029, *p* = 0.0166; and attention was a significant predictor of learning, *b* = 2.3709, *SE* = 1.0839, *p* = 0.0383. Approximately 29% of the variance in learning was accounted for by the predictors (*R*^2^ = 0.2863). Bootstrapping with 5000 samples estimated the indirect effect was significant at the 95% confidence level, *b* = -0.6249, *SE* = 0.3781, CI = -1.5675, -0.0291, supporting the mediational hypothesis.

In both models, book format was no longer a significant predictor of learning after controlling for the mediator (and age and duration), consistent with full mediation (availability: *b* = -0.4897, *SE* = 0.5907, *p* = 0.4143; attention: *b* = -0.4663, *SE* = 0.6364, *p* = 0.4706). However, due to our low power, the null effect supporting full mediation should be interpreted with caution.

## Discussion

In this study, we report differences in parent–child talk and behavior when reading print versus electronic versions of the same books. Children and parents spent twice as much time with the electronic versions of the books in comparison to the traditional print versions. After controlling for this time difference, there was no difference in parents’ content-related, behavioral, or off-topic talk. Thus, contrary to our first hypothesis, and in contrast to prior studies that have been conducted with older children (Chiong et al., 2012; Parish-Morris et al., 2013; Krcmar and Cingel, 2014), parent language did not show the same bias toward behavioral talk when reading electronic books with this younger group. This could be due in part to the simple nature of our electronic books (there were no hotspots for pairs to talk about activating), or the younger age of our children. The only medium-related differences in parental behavior observed were a higher number of utterances dedicated to reading the text with print books, which may be expected due to the automatic narration of the electronic book, and a higher number of parent points to the printed book. This final observation was consistent with our hypothesis and aligns with the parent self-report of higher pointing with print in Strouse and Ganea’s (2017a) survey.

We hypothesized that we would also see less pointing to the electronic book by children, perhaps due to increased touching and control of the device and thus less need to gesture. However, this was not the case; there was a trend in the opposite direction. In addition we observed higher levels of child-initiated content-related comments during the electronic book. Taken together, it appeared that children were very communicative regarding the electronic books, indicating an interest in their content and a desire to share this interest.

We expected overall engagement and positive affect with the electronic books to be higher than with print. Indeed, children paid more attention, displayed more positive affect, and made themselves more available when reading the electronic than the traditional print versions of the books. This is consistent with findings in studies with preschoolers (Moody et al., 2010; Chiong et al., 2012). The emotional quality of the reading interaction and children’s attention and engagement have been linked to future reading motivation and emergent literacy skills (Laakso et al., 1999; Sonnenschein and Munsterman, 2002; Bingham, 2007), suggesting that engagement is an extremely important factor in creating developmentally supportive reading experiences. This, combined with children’s commenting on the book and participation through pointing and page turns, suggests that electronic reading could be a supportive early literacy activity for toddlers, as it is for preschoolers (Takacs et al., 2015).

Contrary to our hypotheses, but adding support to the argument that electronic books support literacy development, children correctly chose a previously unfamiliar animal labeled in the book more often when they had read the electronic than the traditional print book, after controlling for the duration of the reading session. Availability for reading and attention to the book acted as mediators in predicting children’s word learning at test, suggesting that electronic books supported children’s learning by way of increasing their engagement and attention. The current study more accurately reflects children’s typical e-book usage than Strouse and Ganea’s (2017b) word learning study by using a commercially available book and having parents rather than researchers read with children. In their study, the scripted interaction was so heavily controlled by the researchers that natural differences in child attention may not have been able to emerge. The current study suggests that children’s attention and engagement plays an important role in supporting learning from electronic books.

One important factor that must be considered alongside our results is the type of electronic enhancements used in our e-book. The type of multimedia enhancements used may afford different parent and child talk. Our e-book did not incorporate any hotspots for children and parents to activate, which may have partially accounted for low levels of behavioral talk. Chiong et al. (2012) reported that they did not see the same focus on behavioral talk when parents and children were reading plain e-books without enhancements that they did when pairs read books with many hotspots. Similarly, Moody et al. (2010) reported that when the number of hotspots children could activate was restricted that children produced more labels for the items on-screen. As such, the lack of a behavioral focus on the part of our participants is consistent with prior research. However, Krcmar and Cingel (2014) used very simple e-books without enhancements and still reported more behavioral talk when parents read e-books. One possibility is that the simple animations and sound effects present in our stories were well-enough aligned with the content to direct children and parent’s focus on the relevant content of the book. A similar enhancement was reported to maintain the interest of 5-year-olds (Verhallen and Bus, 2009).

Electronic books may also afford different non-verbal behaviors than print. Electronic page turns may be less physically demanding for young children because they require a simple tap rather than a coordinated finger-hand-arm movement. In particular, in our book page turns could be triggered by a tap anywhere on the screen. In addition, allowing children to physically control the book by turning the pages has been suggested as a tool for teaching children the “rules” of reading as part of their developing concept of print (e.g., holding the book upright, reading right to left) (Goodsitt et al., 1988). Children in our study had more prior experience with print than electronic books. As such, it is not surprising that we saw fewer page turns in our print conditions, as children’s concept of print was likely more developed for this medium.

Pointing was marginally more common from children in the electronic conditions and significantly more common from parents in the print conditions. Pointing on the part of the child has been argued to be a communicative behavior in which children direct their parent’s attention or request a label (Murphy, 1978; DeLoache and DeMendoza, 1987), and thus could be indicative of children’s overall engagement with the electronic book. Pointing on the part of the parent has been interpreted as more of a redirection strategy when children have lost attention to the content (Sénéchal et al., 1995), and thus could be indicative of overall lower levels of engagement with the traditional print-format books. In our case, parent pointing to print books did not engage children enough to make attention levels comparable to those with the electronic books.

Besides simple format-based differences, we also found that children with no prior electronic book experience made more content-related comments when reading electronic books than children with prior experience. Krcmar and Cingel (2014) hypothesized that children with experience invested less mental effort in processing the stories because they viewed electronic devices as toys rather than learning tools. They did not report whether parent–child talk in their study differed based on experience, but considering that content-related talk has been associated with comprehension gains from video storybooks (Strouse et al., 2013), increased content-related talk with the e-books could have been one mechanism by which children with lower prior experience could have comprehended their story better. In our study, we did not report a similar effect of prior experience on learning. This may a result of our younger sample, different learning outcome, non-narrative book or other factors. Our sample for the learning analyses was also somewhat limited in size. Future research should probe the relationship between experience, mental effort, behavior, and learning. If experience does lead to lower mental investment and learning, this may become more of an issue as tablet devices become more ubiquitous.

An important limitation to this study is the drop in power we experienced when testing our learning outcomes because of the number of children already familiar with some of the test animals. As a result, we did not have the ability to test the mediating role of parent–child behaviors on learning other than the availability and attention variables. There is a robust literature on parent–child interaction and language and literacy outcomes in preschoolers, but there is very little evidence-based information available about what constitutes high-quality language and actions during reading with toddlers and infants. It is important that before we make value judgments about whether particular formats are supportive of parent–child interaction that we have more information about what exactly high quality parent–child interaction looks like with this age group.

One important caveat to our findings is that increased engagement does not always translate into increased learning. Labbo and Kuhn (2000) called enhancements “considerate” if they relate to the story and give children more detail or information about the story content. “Inconsiderate” enhancements contain extra sounds, animations, or other features that are unrelated to the content and do not assist children in remembering the story. Considerate enhancements have been argued to be particularly supportive of literacy (Labbo and Kuhn, 2000; Turbill, 2001) and used to explain why some studies of electronic books show greater benefits than others (Zucker et al., 2009; Takacs et al., 2015). Thus, while electronic books have the potential to be supportive of language development, certain attributes may make them less effective.

Here, engagement mediated the relationship between book format and learning, but in books of other types or with other learning goals, this may not be the case. Electronic books designed to be interactive through extensive hotspots may have very different story formats and illustrations. Bells and whistles in electronic books can be designed in many ways that may increase children’s participation with them, but if these features do not draw attention to the educational content they may not serve as a supportive feature. For example, Willoughby et al. (2015) reported that 3- and 4-year-olds given the opportunity to interact with electronic alphabet books at school spent more time with them than children who were given print books, but this increased time did not translate to better letter knowledge at post-test. They hypothesized this was likely because children spent their time activating hotspots irrelevant to the letter names or sounds. In addition, experiences activating built-in features that act as entertainment may heighten any tendencies children have to interpret electronic media as games rather than learning tools.

Despite this caveat, it is possible that toddler’s content learning may not suffer from electronic books to the same extent as preschoolers’ learning because toddlers’ books tend to present stand-alone content on each page rather than narrative-based stories. As such, distractions from the content may be less disruptive because children do not need to weave together information across pages. Toddlers may also be used to behavior-related distractions from reading, as book handling is a relatively common part of the reading process at this age. Based on the positive engagement and content-related language we saw in our electronic book group, infants’ and toddlers’ learning from electronic books deserves further study.

Another important limitation of our study is that parent and child behavior in this lab-based observation did not match the behavior reported by parents as typical of their home behavior in Strouse and Ganea’s (2017a) survey. Future research will need to explore whether these differences are due to the location of testing, the type of book read, a mismatch between parent perception and actual behavior, or other factors. Future research should also include samples with a wider variety of socio-economic backgrounds.

In sum, this work extends the prior literature by providing information about toddler–parent experiences reading in different formats. When compared with prior literature it reveals potentially significant age-related differences in the way parents treat digital formats and suggests that much more work is needed to determine the potential benefits and hazards of new media.

## Author Contributions

GS developed the study materials and procedures, collected the data, oversaw the transcription and coding of data, analyzed and interpreted the data, and wrote the manuscript. PG provided guidance in the development of the project and edited the manuscript.

## Conflict of Interest Statement

The authors declare that the research was conducted in the absence of any commercial or financial relationships that could be construed as a potential conflict of interest.

## Footnotes

1 Half of the parents in each experimental condition were asked to read “as they would if they had this book at home” and half were asked to “use this book to teach your child any of the animal names he/she does not already know.” This manipulation was initially included in our analyses, however, no patterns important to the hypotheses or the statistical models reported in this paper emerged, so we collapsed these groups and this instruction is not discussed further.

2 http://talkbank.org/clan/

3 http://datavyu.org

4 http://processmacro.org/download.html
