# Supplementary material for: Parent–Toddler Behavior and Language Differ When Reading Electronic and Print Picture Books
Source: Front Psychol. 2017 May 16;8:677. doi: 10.3389/fpsyg.2017.00677 (PMC5432581; doi:10.3389/fpsyg.2017.00677)

Table 1

Predictors of Reading Duration.

Fixed effects	df	F	p
Intercept	1, 89.86	19.01	<.001***
Book format	**1, 88.44**	**74.70**	**<.001*** **
Prior experience	1, 88.39	2.82	.097
Book format x experience	1, 88.39	1.91	.170
Book content	1, 91.88	2.52	.116
Book format x content	1, 91.88	19.21	<.001***
Age	1, 89.65	2.13	.148

Note. $^{*}p < .05$. $^{**}p < .01$. $^{***}p < .001$.

Table 2

Predictors of Child Non-Verbal Behaviours

	Pointing			Page turns		
Fixed effects	df	F	p	df	F	p
Intercept	1, 93.75	0.02	.877	1, 97.18	0.06	.815
Book format	1, 115.13	3.62	.060	**1, 117.27**	**4.42**	**.038***
Prior experience	1, 88.42	0.76	.385	1, 89.92	0.07	.792
Book format x experience	1, 86.81	1.83	.179	1, 90.27	0.03	.873
Book content	1, 90.12	0.98	.326	1, 92.70	0.59	.445
Book format x content	1, 95.68	0.24	.626	1, 97.60	0.86	.357
Age	1, 87.96	0.89	.348	1, 91.22	2.10	.150
Duration	1, 177.94	2.91	.090	1, 169.45	0.04	.840

Note. $^{*}p < .05$. $^{**}p < .01$. $^{***}p < .001$.

Table 3

Predictors of Child Language Content

	Content-related comments			Behavior-related talk		
Fixed Effects	df	F	p	df	F	p
Intercept	1, 103.06	.073	.395	1, 102.07	.022	.641
Book format	**1, 125.03**	**6.97**	**.009****	1, 123.19	3.61	.060
Prior experience	1, 96.21	1.60	.209	1, 95.84	2.70	.104
Book format x experience	**1, 95.76**	**3.96**	**.049***	1, 95.41	1.74	.190
Book content	1, 100.70	9.40	.003**	1, 100.863	4.18	.043*
Book format x content	1, 106.60	6.93	.010*	1, 106.94	1.15	.287
Age	1, 97.57	0.42	.521	1, 97.32	1.72	.193
Duration	1, 186.07	0.29	.588	1, 179.74	10.04	.002**

Note. $^{*}p < .05$. $^{**}p < .01$. $^{***}p < .001$.

Table 4

Predictors of Parent Non-Verbal Behaviours

	Points			Page turns		
Fixed Effects	df	F	p	df	F	p
Intercept	1, 99.79	5.82	.018*	1, 99.59	1.01	.316
Book format	1, 119.75	0.75	.390	**1, 118.88**	**15.40**	**<.001*****
Prior experience	1, 92.52	1.83	.180	1, 92.46	0.41	.526
Book format x experience	1, 92.87	0.42	.518	1, 92.78	0.01	.915
Book content	1, 95.38	1.36	.246	1, 94.85	0.36	.553
Book format x content	1, 100.31	1.21	.275	1, 99.44	0.56	.457
Age	1, 93.94	0.98	.326	1, 93.66	0.04	.835
Duration	1, 170.91	12.69	<.001***	1, 165.55	9.07	.003**

Note. $^{*}p < .05$. $^{**}p < .01$. $^{***}p < .001$.

Table 5

Predictors of Parent Language

	Content-related talk			Behavior-related talk			Off-topic talk		
Fixed Effects	df	F	p	df	F	p	df	F	p
Intercept	1, 101.83	14.72	<.001***	1, 103.56	1.17	.282	1, 102.04	0.42	.518
Book format	1, 118.98	1.78	.185	1, 126.00	0.13	.716	1, 124.01	0.56	.457
Prior experience	1, 95.25	0.04	.851	1, 96.13	1.11	.294	1, 95.24	0.02	.878
Book format x experience	1, 94.93	3.19	.078	1, 95.68	0.02	.879	1, 94.80	0.56	.455
Book content	1, 96.01	0.85	.358	1, 99.90	0.12	.731	1, 99.80	0.00	.995
Book format x content	1, 99.1	8.64	.004**	1, 105.38	1.94	.166	1, 105.73	2.45	.120
Age	1, 95.69	3.30	.072	1, 97.30	1.34	.250	1, 96.62	0.05	.829
Duration	1, 150.16	35.41	<.001***	1, 188.98	20.25	<.001***	1, 185.44	43.91	<.001***

Note. $^{*}p < .05$. $^{**}p < .01$. $^{***}p < .001$.

Table 6

Predictors of Children's Attention.

Fixed Effects	df	F	p
Intercept	1, 93.67	45.48	<.001***
Book format	**1, 120.95**	**21.78**	**<.001*** **
Prior experience	1, 89.15	0.23	.634
Book format x experience	1, 89.37	1.51	.223
Book content	1, 86.29	1.37	.246
Book format x content	1, 93.25	2.43	.122
Age	1, 86.44	15.60	<.001***
Duration	1, 167.07	30.38	<.001***

Note. $^{*}p < .05$. $^{**}p < .01$. $^{***}p < .001$.

Table 7

Predictors of Children's Availability for Reading, Affect, and Participation

	Availability			Affect			Participation		
Fixed effects	df	F	p	df	F	p	df	F	p
Intercept	1, 97.45	32.94	<.001***	1, 97.61	65.59	<.001***	1, 97.22	10.19	.002
Book format	**1, 116.10**	**17.60**	**<.001*** **	**1, 113.78**	**12.85**	**<.001*** **	1, 113.72	1.32	.253
Prior experience	1, 90.28	0.01	.946	1, 91.07	0.93	.338	1, 90.58	0.13	.720
Book format x experience	1, 90.39	0.00	.989	1, 91.15	1.28	.262	1, 90.67	0.45	.505
Book content	1, 92.94	0.28	.596	1, 92.50	1.18	.281	1, 92.14	4.78	.031*
Book format x content	1, 96.87	0.16	.695	1, 96.07	.0.51	.476	1, 95.81	0.19	.666
Age	1, 91.94	16.66	<.001***	1, 91.98	8.57	.004	1, 91.52	7.18	.009**
Duration	1, 168.23	17.16	<.001***	1, 150.60	5.07	.02	1, 152.29	7.34	.008**

Note. $^{*}p < .05$. $^{**}p < .01$. $^{***}p < .001$.

Table 8

Predictors of Children's Learning

		Model 1		Model 2	
Fixed effects	df	Wald χ2	p	Wald χ2	p
Intercept	1	0.04	.851	0.73	.394
Book format	1	**7.36**	**.007****	2.38	.123
Prior experience	1	0.87	.351	3.08	.079
Book format x experience	1	2.61	.106	0.19	.661
Test trials	2	1.29	.525	.333	.847
Experimental vs control	*1*	*n/a*	*n/a*	0.00	.987
Familiar animal choices	1	6.73	.009**	2.18	.140
Age	1	0.93	.334	0.46	.498
Duration	1	5.35	.021*	*n/a*	*n/a*

Note. Model 1 produced a better fit, QICC = 141.32, than model 2, QICC = 233.86

$^{*}p < .05$. $^{**}p < .01$. $^{***}p < .001$.

Figure 1. Z-scores were calculated for the displayed variables. The average z-score for children reading each book format is displayed. Error bars represent the standard error of the z-scores. Asterisks represent the significance of the main effect of book format after adjusting for covariates, as reported in the text. $^{*}p < .05$. $^{**}p < .01$. $^{***}p < .001$.

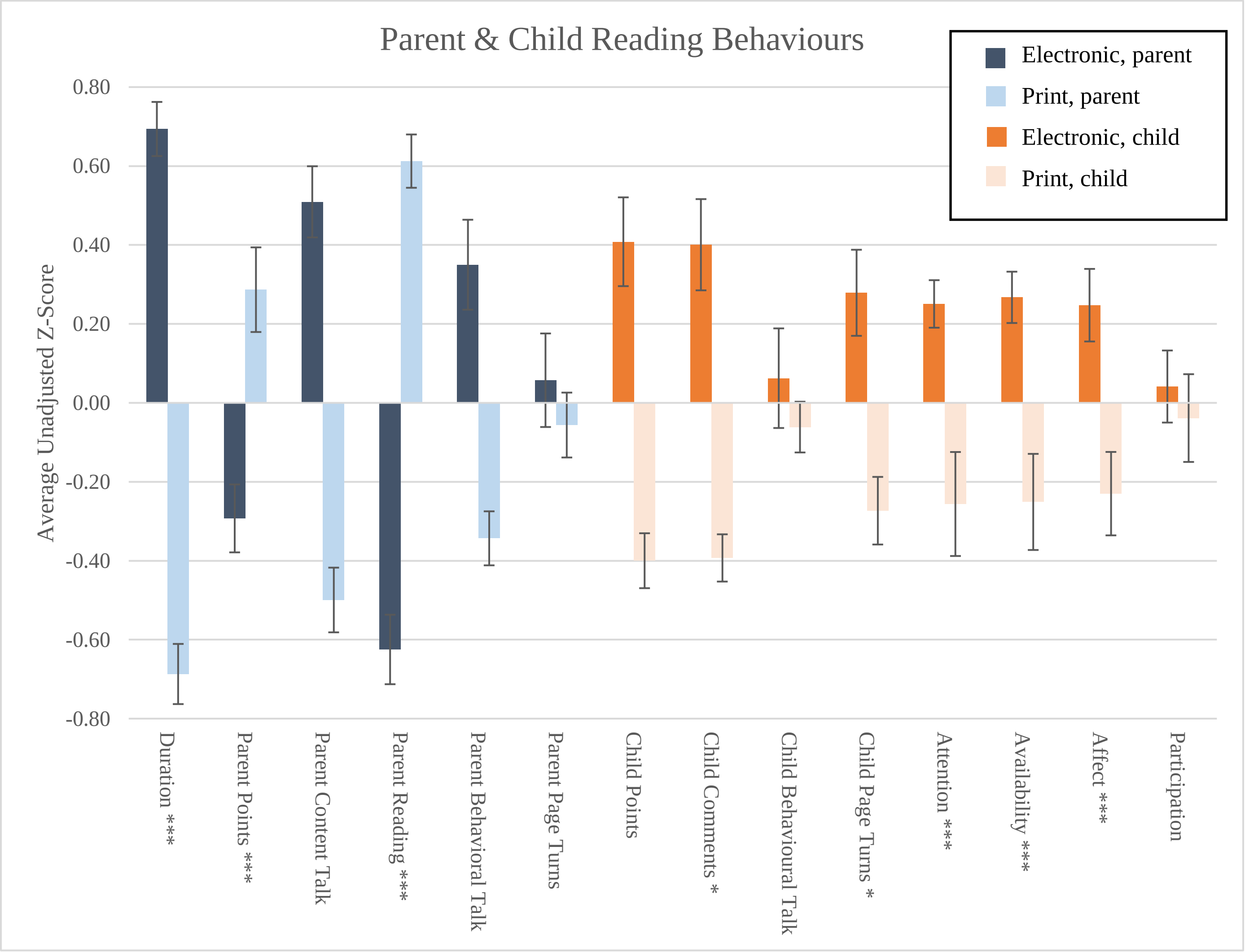

Supplement: Supplementary file 1 [file Data_Sheet_1.pdf]
